# miR482 Regulation of *NBS-LRR* Defense Genes during Fungal Pathogen Infection in Cotton

**DOI:** 10.1371/journal.pone.0084390

**Published:** 2013-12-31

**Authors:** Qian-Hao Zhu, Longjiang Fan, Yang Liu, Hao Xu, Danny Llewellyn, Iain Wilson

**Affiliations:** 1 CSIRO Plant Industry, Canberra, ACT, Australia; 2 Department of Agronomy, Zhejiang University, Hangzhou, China; New Mexico State University, United States of America

## Abstract

In this study, we characterized the miR482 family in cotton using existing small RNA datasets and the recently released draft genome sequence of *Gossypium raimondii*, a diploid cotton species whose progenitor is the putative contributor of the D_t_ (representing the D genome of tetraploid) genome of the cultivated tetraploid cotton species *G. hirsutum* and *G. barbadense*. Of the three ghr-miR482 members reported in *G. hirsutum*, ghr-miR482a has no homolog in *G. raimondii*, ghr-miR482b and ghr-miR482c each has a single homolog in *G. raimondii*. Gra-miR482d has five homologous loci (gra-miR482d, f-i) in *G. raimondii* and also exists in *G. hirsutum* (ghr-miR482d). A variant, miR482.2 that is a homolog of miR2118 in other species, is produced from several *GHR-MIR482* loci in *G. hirsutum*. Approximately 12% of the *G. raimondii NBS-LRR* genes were predicted targets of various members of the gra-miR482 family. Based on the rationale that the regulatory relationship between miR482 and *NBS-LRR* genes will be conserved in *G. raimondii* and *G. hirsutum*, we investigated this relationship using *G. hirsutum* miR482 and *G. raimondii NBS-LRR* genes, which are not currently available in *G. hirsutum*. Ghr-miR482/miR482.2-mediated cleavage was confirmed for three of the four *NBS-LRR* genes analysed. As in tomato, miR482-mediated cleavage of *NBS-LRR* genes triggered production of phased secondary small RNAs in cotton. In seedlings of the susceptible cultivar Sicot71 (*G. hirsutum*) infected with the fungal pathogen *Verticillium dahliae*, the expression levels of ghr-miR482b/miR482b.2, ghr-miR482c and ghr-miR482d.2 were down-regulated, and several *NBS-LRR* targets of ghr-miR482c and ghr-miR482d were up-regulated. These results imply that, like tomato plants infected with viruses or bacteria, cotton plants are able to induce expression of *NBS-LRR* defence genes by suppression of the miRNA-mediated gene silencing pathway upon fungal pathogen attack.

## Introduction

microRNAs (miRNAs) are 20–24 nucleotides (nt) long small noncoding RNAs and are processed from *MIRNA* genes that are transcribed by RNA polymerase II. In plants, the primary transcript (pri-miRNA) of a *MIRNA* gene is processed by the RNase III-like enzyme DICER-LIKE1 (DCL1) into a hairpin structure, miRNA precursor or pre-miRNA, which is further cleaved by DCL1 to produce a miRNA/miRNA* duplex from the stem region of the hairpin. The miRNA/miRNA* duplex is assembled into a RNA induced silencing complex (RISC). Once incorporated into the RISC, the mature miRNA binds to complementary sites in its target genes through base pairing and causes degradation and/or translational repression of the target mRNAs, which depends on the level of complementarity between miRNA and its targets [1]. miRNAs are posttranscriptional repressors of gene activity; however, depending on the role of their target genes, they can be positive or negative regulators of a certain biological process. Since the discovery of miRNAs in plants a decade ago, miRNAs have been demonstrated to play a critical role in many aspects of plant development, biological processes and stress responses [2], [3], [4], [5], [6].

Pathogen infection triggers transcriptional reprogramming in host plants. Plants have evolved multiple layers of mechanisms to protect themselves from pathogen attacks, including non-host specific resistance, PAMP (pathogen-associated molecular pattern)-trigged immunity (PTI) and effector-triggered immunity (ETI) [7]. A number of miRNAs in *Arabidopsis* have been shown to either positively or negatively regulate elicitor flg22-induced callose deposition, a signature of the PTI defense response [8]. miR393 has been implicated in bacterial PTI through repressing auxin signaling [9]. *Arabidopsis* plants elicited by flg22 showed induction of *MIR393* transcription and down-regulation of miR393 targets, including three F-box auxin receptors *TIR1* (*Transport Inhibitor Response 1*), *AFB2* (*Auxin signaling F-Box proteins 2*) and *AFB3*, and consequently increased resistance to *Pseudomonas syringae* [9]. More recently, miRNAs have been shown to be directly involved in regulation of disease resistance (*R*) genes [10], [11], [12]. The *N* gene from *Nicotiana benthamiana* [13], which encodes a TIR (the Toll and Interleukin-1 Receptor) type of nucleotide binding site (NBS)-leucine-rich-repeat (LRR) receptor protein that confers resistance to tobacco mosaic virus (TMV), was found to be cleaved by nta-miR6019 and nta-miR6020 [11]. Transient expression of *N*-targeted miRNAs in *N. benthamiana* attenuates *N*-mediated resistance to TMV [11], indicating that nta-miR6019 and nta-miR6020 play an important role in regulating disease resistance in *N. benthamiana*. Further bioinformatic mining identified several miRNAs, including miR482 and miR2118, which target members of different *R*-gene families in tomato, potato, soybean and *Medicago truncatula* [10], [11]. miR482 and miR2118 are partially overlapping and both were predicted to target the conserved sequences encoding the P-loop motif of the NBS-LRR receptors [10], [11], [12], so would be expected to suppress the expression of large numbers of *NBS-LRR* defense genes. Using a transient assay system, it has been shown that *N. benthamiana* mRNAs encoding NBS-LRR proteins could be silenced by tomato miR482 [12].

miR482, miR2118 and nta-miR6019 belong to a specific type of miRNA that is 22-nt long and generated from pre-miRNAs containing asymmetric bulges in the miRNA/miRNA* duplex. This type of miRNA has been demonstrated to be the trigger for production of phased 21-nt secondary small RNAs from their target transcripts through the RDR6/DCL4 pathway [14], [15]. Therefore, miR482-, miR2118- and nta-miR6019-mediated cleavage of target disease resistance genes is expected to cause not only decay of their target mRNAs but also production of phased secondary small RNAs from the targeted *R*-genes. This has been verified in tomato and *M. truncatula* [10], [11], [12]. Furthermore, at least one of the secondary small RNAs generated from a miR482 targeted *NBS-LRR* gene has been shown to target mRNA encoding another defense-related protein [12], indicating that the secondary small RNAs generated from *NBS-LRR* genes can possess the characteristic of trans-acting siRNAs (ta-siRNAs) [16], [17]. Plants infected with viruses or bacteria showed a reduced level of miR482 and an increased level of mRNAs of miR482 targets, suggesting that the miR482-mediated silencing cascade is suppressed by pathogen attack and may be a defense response of plants [12].

Cotton is the most important textile fiber crop in the world. The cotton genus (*Gossypium* spp.) consists of 50 species, including 45 diploids and five allotetraploids. Two A-genome species (*G. arboreum* and *G. herbaceum*) and two AD-genome species (*G. hirsutum* and *G. barbadense*) were independently domesticated and cultivated for their fibers [18]. All the allotetraploids originated from relatively recent interspecific hybridization events between an A-genome-like ancestral African species similar to modern *G. arboreum* or *G. herbaceum* and a D-genome-like Central American species similar to modern *G. raimondii* [19]. Because of the importance of fiber, miRNA identification in cotton has mainly focused on fiber tissues, particularly tissues related to fiber initiation [20], [21], [22], although other tissues such as embryogenic callus have been used [23]. In the miRBase v20 (updated in June 2013), only 86 cotton miRNAs have been annotated (80 in *G. hirsutum*, one each in *G. arboreum* and *G. herbaceum*, and four in *G. raimondii*). This paucity is largely due to the lack of cotton genome sequences and use of only a narrow range of tissues in miRNA identification. This has changed with the release of the draft genome assembly of *G. raimondii*, a D-genome diploid cotton species that is the closest living relative of the ancestral D_t_-genome in tetraploid cotton [24], [25], from which 28 conserved and 181 non-conserved miRNA families were predicted [24]. Another study investigated cotton miRNAs responsive to *Verticillium dahliae* infection [26]. *V. dahliae* is a fungal pathogen causing the soil-borne vascular disease verticillium wilt (VW), one of the major cotton diseases worldwide. The small RNA populations in *V. dahliae*-inoculated cotton (*G. hirsutum* and *G. barbadense*) roots were sequenced and compared with those from mock-treated roots. This investigation identified a number of conserved miRNAs, including miR2118, and 14 new cotton miRNAs, which were up- or down-regulated upon *V. dahliae* infection [26].

In this study, we characterized the cotton miR482 family using published small RNA datasets [10], [26] and the genome sequence of *G. raimondii* [24], identified *G. raimondii NBS-LRR* genes potentially targeted by miR482/miR482.2, and analyzed *V. dahliae*-induced expression changes of miR482/miR482.2 and their predicted *NBS-LRR* targets in *G. hirsutum*. We found that about 12% of the *G. raimondii NBS-LRR* genes are potential targets of miR482/miR482.2, and that miR482-mediated cleavage of *NBS-LRR* genes triggers production of phased secondary small RNAs. Four members of the ghr-miR482 family and several *NBS-LRR* genes were down-regulated and up-regulated in cotton seedlings infected with *V. dahliae*, respectively, implying that miR482-mediated silencing of *NBS-LRR* genes is released in cotton upon fungal pathogen infection to activate disease defense.

## Results

### The Cotton miR482 Family

**Figure 1:**
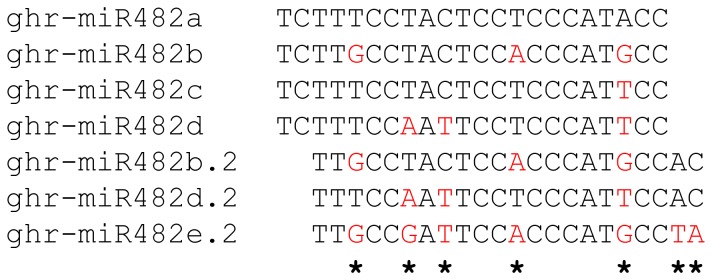
Alignment of *G. hirsutum* miR482/miR482.2 members. * indicates the positions with variable nucleotides.

The first member of the cotton miR482 family was identified in *G. hirsutum* [20]. Three more members of this family, ghr-miR482a and ghr-miR482b in *G. hirsutum* and gra-miR482 in *G. raimondii*, were later reported [21]. By searching the published cotton small RNA datasets, we found that gra-miR482 also exists in *G. hirsutum*; therefore, there are at least four miR482 members in the *G. hirsutum* genome. In order to characterize the cotton miR482 family and keep the original nomenclature as much as possible, we renamed the first miR482 identified by Kwak et al. [20] as ghr-miR482c and the *G. hirsutum* counterpart of gra-miR482 as ghr-miR482d (Figure 1). Ghr-miR472 reported by Kwak et al. [20] is in fact ghr-miR482a. The miR482 reported by Wang et al. [22] is a variant of ghr-miR482b because its 1^st^ – 20^th^ nucleotides are identical to the 3^rd^ – 22^nd^ nucleotides of ghr-miR482b (Figure 1). We designated it ghr-mi482b.2 (similar to miR2118 in other plant species). Using ghr-miR482b.2 as a query and searching against *G. hirsutum* small RNA datasets, we found two isoforms of ghr-miR482b.2. One is identical to ghr-miR482d except for the last two nucleotides; another has no corresponding ghr-miR482 sequence found in *G. hirsutum*. We named these two as ghr-miR482d.2 and ghr-miR482e.2, respectively (Figure 1). We found no similar variants for ghr-miR482a and ghr-miR482c in *G. hirsutum*.

**Figure 2:**
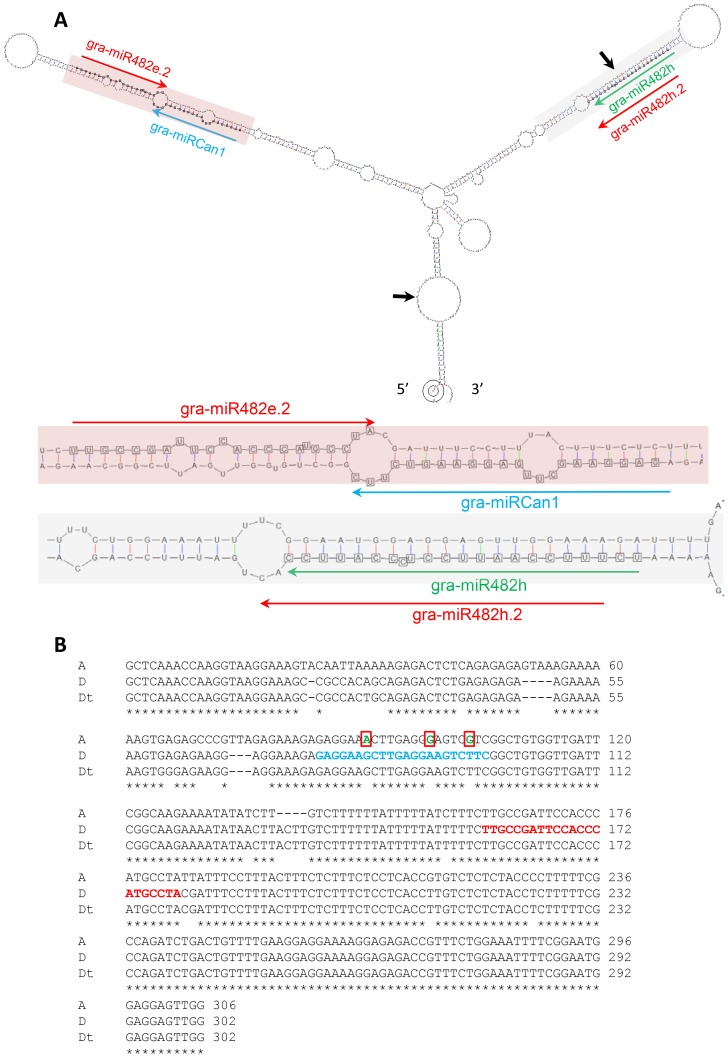
A single primary *MIRNA* transcript contains gra-miR482h/miR482h.2 and gra-miR482e.2. A) Hairpin structure of the precursor (*G. raimondii*) containing gra-miR482h/miR482h.2 and gra-miR482e.2. The details of the shaded regions of the precursor are shown below the hairpin structure. B) Aligned sequence fragments from the A genome (*G. arboreum* var. YZ) and the D_t_ genome (*G. hirsutum* var. TM-1) corresponding to the D genome (*G. raimondii*) indicated by two black arrows in A. Amplification of the A_t_ genomic fragment from *G. hirsutum* failed probably due to sequence difference between the A_t_ and D_t_ genome at the primer annealing regions. miRCandidate1 (miRCan1) and miR482e.2 are highlighted in blue and red, respectively. Three SNPs that abolished the production of miRCan1 in the A genome are boxed.

To determine whether these *G. hirsutum* miRNAs exist in the *G. raimondii* genome, and how many loci are able to generate these miRNAs in the *G. raimondii* genome, we searched the *G. raimondii* genome for sequences that exactly match with ghr-miR482 and predicted hairpin structure using the genomic sequence surrounding the miRNA. We found that the *G. raimondii* genome contains a single locus each for gra-miR482b and gra-miR482c. The sequence identical to ghr-miR482d is detected at five loci that are able to form a stem-loop structure in the *G. raimondii* genome. They were named as gra-miR482d, f, g, h, and i (Figure 2A; Figure S1). No homolog of ghr-miR482a was found in the *G. raimondii* genome. Correspondingly, gra-miR482b.2, gra-miR482d.2, gra-miR482f.2, gra-miR482g.2, gra-miR482h.2, and gra-miR482i.2 as well as gra-miR482e.2 were found in the *G. raimondii* genome. When allowing one mismatch, we found five more loci each containing a gra-miR482d isoform and predicted to form a hairpin structure in the *G. raimondii* genome (Figure S2); however, these new gra-miR482d isoforms were not found in any of the published cotton (*G. hirsutum*, *G. barbadense* and *G. arboreum*) small RNA datasets so are not further analysed.

### Gra-miR482h/miR482h.2 and Gra-miR482e.2 are Tandem miRNAs

Both gra-miR482h/miR482h.2 and gra-miR482e.2 are located on scaffold 9 of *G. raimondii*, and are just 164 bp apart from each other. The *G. raimondii* genomic sequence containing these two miRNAs is predicted to form two hairpins. Gra-miR482h/miR482h.2 and gra-miR482e.2 are located within each of these two hairpins (Figure 2A). Because these two miRNAs are closely located, they could be generated from the same primary transcript. A gene model (Gorai.009G338400, no potential function annotated) is predicted in the region containing gra-miR482h/miR482h.2 and gra-miR482e.2 in the *G. raimondii* genome although it has no homologous gene in other plants so is presumably non-coding. By searching the cotton gene index (DFCI) database (http://compbio.dfci.harvard.edu/cgi-bin/tgi/gimain.pl?gudb=cotton), we found a tentative consensus sequence TC257237 (1446 bp in length), which was assembled from a few *G. raimondii* expressed sequence tags (ESTs) and is fully reverse complementary to the *G. raimondii* genomic sequence containing gra-miR482h/miR482h.2 and gra-miR482e.2. This finding suggests that TC257237 is likely to be the primary transcript for gra-miR482h/miR482h.2 and gra-miR482e.2 and that its orientation is probably mis-annotated in the DFCI database (Figure S3).

By mapping *G. hirsutum* small RNAs to the *G. raimondii* sequence containing gra-miR482h/miR482h.2 and gra-miR482e.2, we found that the hairpin harbouring gra-miR482e.2 contains two phased miRNA/miRNA* duplexes, one is gra-miR482e.2/miR482e.2* and another is gra-miRCan1/miRCan1* (the small RNA with more reads was designated gra-miRCan1; Figure 2A). This second miRNA appears to be unique to the D and D_t_ genomes as when we cloned the same region from diploid and tetraploid cotton species the miRCan1/miRCan1* duplex was only found in the D (*G. raimondii*) and D_t_ (*G. hirsutum*) genomes (Figure S4). In comparison to the D and D_t_ genome sequences, the A genome (*G. arboreum*) sequence contains three single nucleotide polymorphisms (SNPs) in miRCan1, which would abolish the base pairing between miRCan1 and miRCan1* and change the hairpin structure of the A genome sequence (Figures 2B, S4). Two potential targets, including a gene encoding a leucine-rich repeat kinase, were predicted for gra-miRCan1 (Table S1).

### *NBS-LRR* Defense Genes are Targets of miR482 in Cotton

In tomato and *M. truncatula*, miR482 and miR2118 have been shown to regulate numerous *NBS-LRR* defense genes through targeting the conserved sequences encoding the P-loop motif of the NBS-LRR receptors [10], [12]. In cotton, only one EST (NP673173) encoding an NBS domain protein has been predicted to be a target of ghr-miR482a/miR482b [21]. This is because no NBS-LRR receptor encoding genes have been reported and only a very limited number of disease resistance related ESTs are available in cotton, although partial genomic sequences for a number of *NBS-LRR* gene analogues have been amplified in both diploid and allotetraploid cottons based on the sequence information of the conserved motifs in other plant species [27], [28], [29].

**Figure 3:**
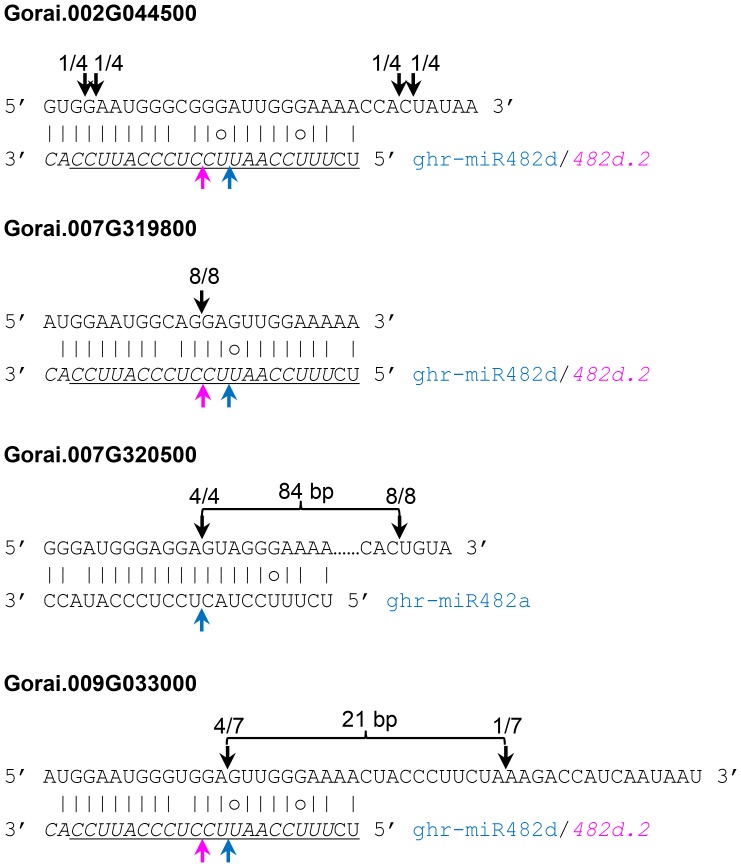
Ghr-miR482/miR482.2-mediated cleavage of *NBS-LRR* genes. Cleavage analysis was performed using 5′ RACE. Two bands were amplified and sequenced for *Gorai.007G320500* whereas a single band was sequenced for other three genes. *Gorai.007G320500* is also a predicted target of ghr-miR482c. The sequences of ghr-miR482d and ghr-miR482d.2 are underlined and showed in italic, respectively. The expected cleavage sites of ghr-miR482 and ghr-miR482.2 were indicated by pink and blue arrows, respectively. The identified cleavage sites are indicated by black arrows with the frequency of cleavage showing on top of the arrows.

Release of the *G. raimondii* genome sequence provided us the opportunity to estimate the number of *NBS-LRR* genes in cotton and how many are targets of miR482. According to the results reported by Paterson et al. [24], the *G. raimondii* genome contains 300 *NBS-LRR* genes. We found that 36 (12%) of these 300 *NBS-LRR* genes were potentially targeted by various numbers of the gra-miR482 (or ghr-miR482) family members. Of these 36 candidate *NBS-LRR* targets, 16 were uniquely targeted by only one member of the gra-miR482 family and 20 were targeted by at least two members (Table S1). Of the members of the gra-miR482 family, gra-miR482b/miR482b.2 had fewer *NBS-LRR* targets than other members (Table S1). Four predicted targets of ghr-miR482/miR482.2 were selected for cleavage analysis using the approach of rapid amplification of cDNA ends or 5′ RACE. *Gorai.007G320500* and *Gorai.009G033000* were confirmed to be targets of ghr-miR482 whereas *Gorai.007G319800* was found to be targeted by ghr-miR482d.2. For *Gorai.002G044500* the cleavage sites were not found at the expected positions but at their nearby positions (Figure 3).

**Figure 4:**
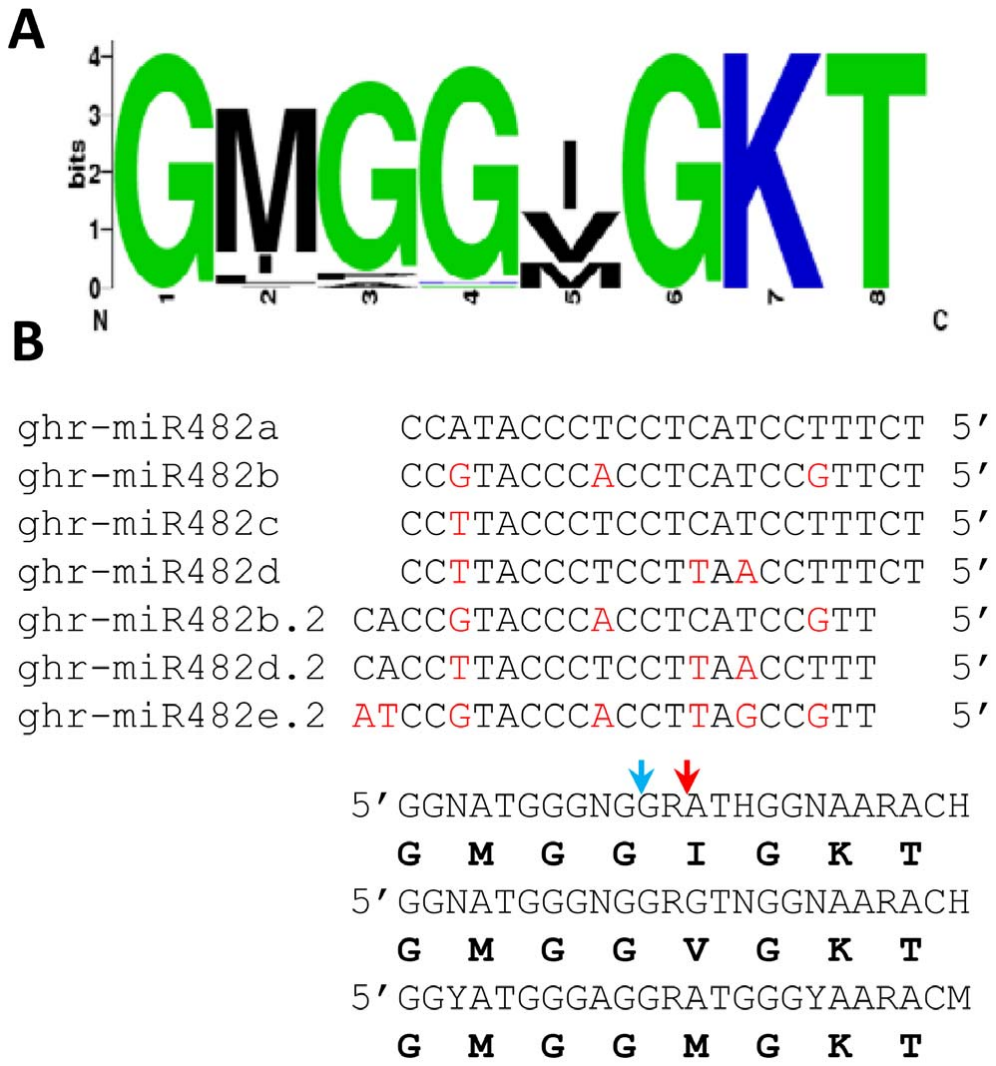
P-loops of the predicted targets of ghr-miR482/miR482.2 in cotton (*G. raimondii*). A) The P-loop sequence logo generated using all predicted *NBS-LRR* targets of ghr-miR482/miR482.2 in *G. raimondii*. B) Top three types of consensus nucleotide sequences of the predicted ghr-miR482/miR482.2 targets and their alignments with ghr-miR482/miR482.2. The corresponding amino acid sequences (P-loop) are shown underneath each consensus nucleotide sequence. The red and blue arrows indicate the expected cleavage position of ghr-miR482 and ghr-miR482.2, respectively. N = A or C or G or T; R = A or G; H = A or C or T; Y = C or T; M = A or C.

The P-loop sequence of the *NBS-LRR* genes targeted by members of the ghr-miR482 family is shown in Figure 4A. It is clear that the last three amino acids GKT that complement the first seven nucleotides of ghr-miR482 were completely conserved in all predicted *NBS-LRR* targets. According to the consensus nucleotide sequences of the top three P-loops that were found in the majority of *NBS-LRR* genes targeted by ghr-miR482, a variable nucleotide was always observed at the 3^rd^ position of a codon (Figure 4B). These characteristics of the target sites suggest that gain and loss of regulation of *NBS-LRR* genes by ghr-miR482 is possible without changes to the protein sequence. In addition, several genes with a diverse function, including a gene encoding a MYB-domain containing protein, were also predicted to be targeted by member(s) of the miR482 family (Table S1).

### Generation of phased Secondary Small RNAs in *NBS-LRR* Genes

**Figure 5:**
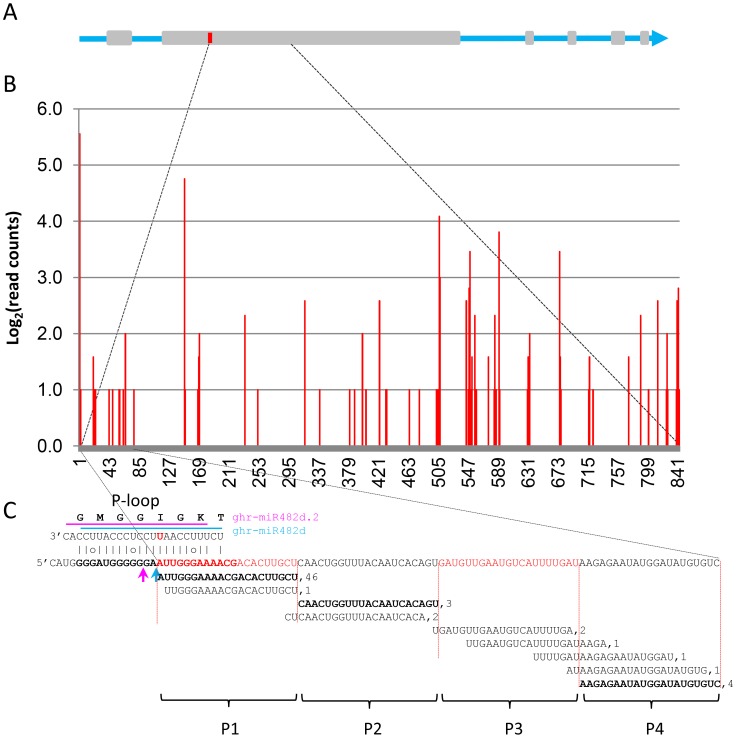
Ghr-miR482d trigged production of phased secondary small RNAs in *Gorai.011G075600*. A) Schematic diagram of *Gorai.011G075600*. Grey boxes represent exons. The red bar represents the location of the P-loop of the NBS domain. B) Distribution pattern of small RNAs generated from the region immediately after the P-loop, which was cleaved by ghr-miR482d. C) Genomic sequence of the P-loop and the first four 21-nt phases (P1–P4). All small RNAs generated from the region corresponding to phases P1 to P4 are shown with the small RNAs in precise phased registers shown in bold. The number after each small RNA represents the counts of the corresponding small RNA in the small RNA datasets used in this study. The sequences underneath the pink and blue lines are ghr-miR482d and ghr-miR2118d, respectively. The expected cleavage sites of ghr-miR482d and ghr-miR2118d are indicated by blue and pink arrow, respectively.

To further confirm the authenticity of the predicted targets and to know whether phased secondary small RNAs were generated from the cleaved targets, we mapped four publically available cotton small RNA datasets (GSM699074-GSM699077 from *V. dahliae*-infected and mock-treated roots of *G. hirsutum* and *G. barbadense*) onto the *G. raimondii* genome and analysed the distribution of small RNAs across all the predicted targets of ghr-miR482/miR482.2 using the method previously described [17], [30]. Of the 36 candidate *NBS-LRR* targets, the majority had small RNAs mapped to, and nine showed a phased distribution of those small RNAs (phase score >2) spreading downstream from the primary target site of ghr-miR482/miR482.2 (Figures 5, S5). Distribution of small RNAs from the first four phases after the ghr-miR482d cleavage site in *Gorai.011G075600* is shown in Figure 5. In this case, the 5' end of the first phased 21-nt small RNA was aligned with the expected cleavage site (the 10^th^ nucleotide counting from the 5' end) of ghr-miR482d rather than that of ghr-miR482d.2, suggesting that production of the phased small RNAs was triggered by ghr-miR482d-mediated cleavage. 21-nt small RNAs corresponding to the first phased register of ghr-miR482 were also observed in *Gorai.007G320300* and *Gorai.007G320500* although no phase signal was bioinformatically detected for these two genes probably because the number of phased small RNAs were below the threshold. For *Gorai.007G320500*, ghr-miR482-mediated cleavage was also confirmed by cleavage analysis (Figure 3). For *Gorai.007G320500* and *Gorai.009G033000*, apart from the ghr-miR482-mediated cleavage, additional cleavage sites located downstream and in phase with the cleavage sites of ghr-miR482d were found (Figure 3), further suggesting that these genes behave like *TAS* loci in *Arabidopsis*.

### Expression Changes of miR482 and *NBS-LRR* Genes upon *V. dahliae* Infection

**Figure 6:**
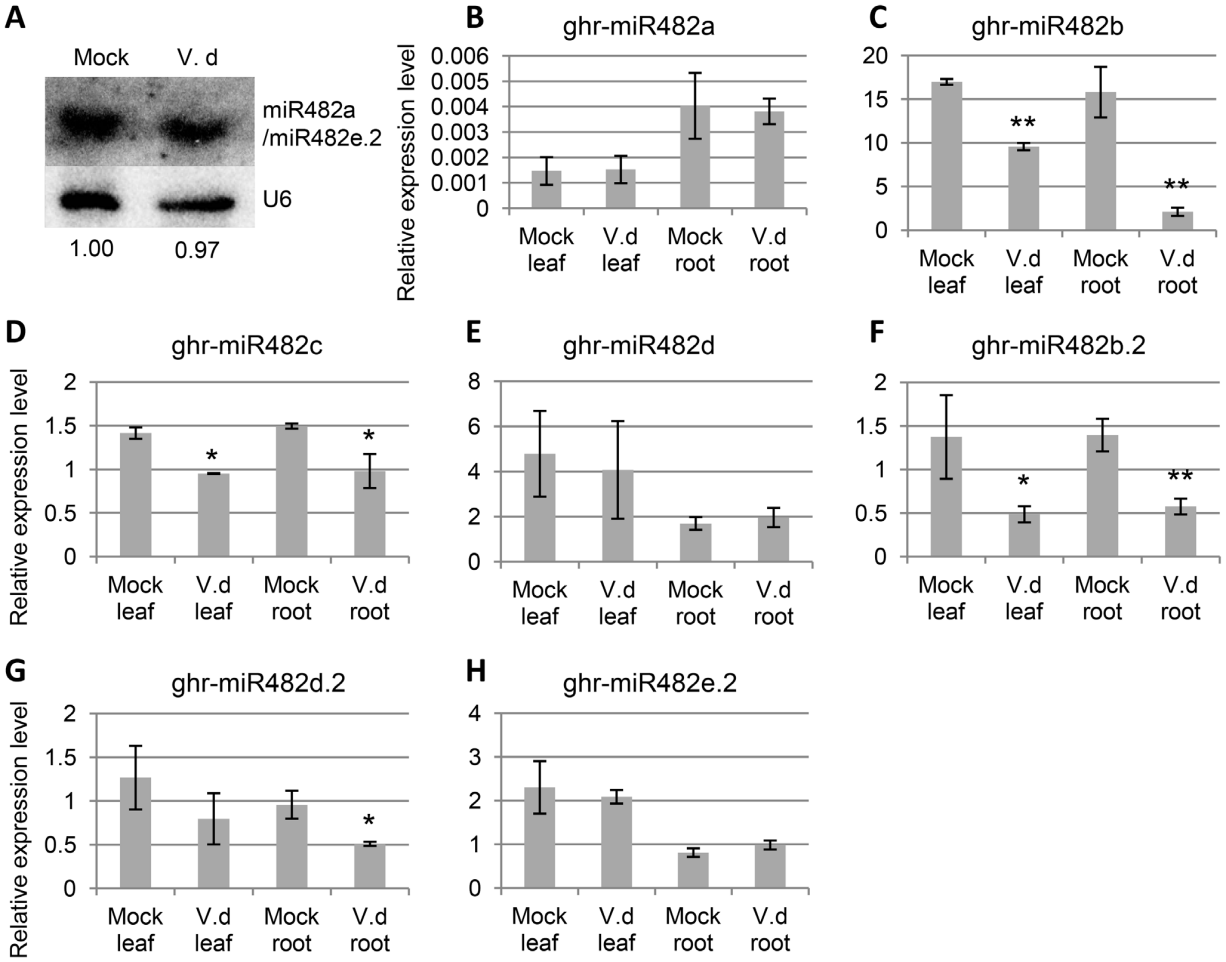
Expression analysis of ghr-miR482/miR482.2. A) Northern blot detection of ghr-miR482/482.2 in leaves collected from *V. dahliae*-infected and mock-treated cotton plants at one day-post-inoculation (1 dpi). Oligos antisense to ghr-miR482a and ghr-miR482e.2 were used together as probes. The values underneath the image are the relative signal intensity of ghr-miR482a/482e.2, which were determined using the MultiGauge V2.0 (Fuji Film) and normalized based on U6. V.d: *V. dahliae*-infected. B to H) Stem-loop qRT-PCR analysis of the expression level of individual members of the ghr-miR482 family. RQ1 DNase treated total RNA isolated from leaves and roots of 1-dpi plants was analysed using ghr-miR482/miR482.2 member specific stem-loop RT and PCR primers. Expression level was normalized to reference gene Histone 3. Error bars represent standard deviation of the expression ratio. * and ** denote significant relative to the corresponding mock-infected control at *p*<0.05 and *p*<0.01, respectively.

**Figure 7:**
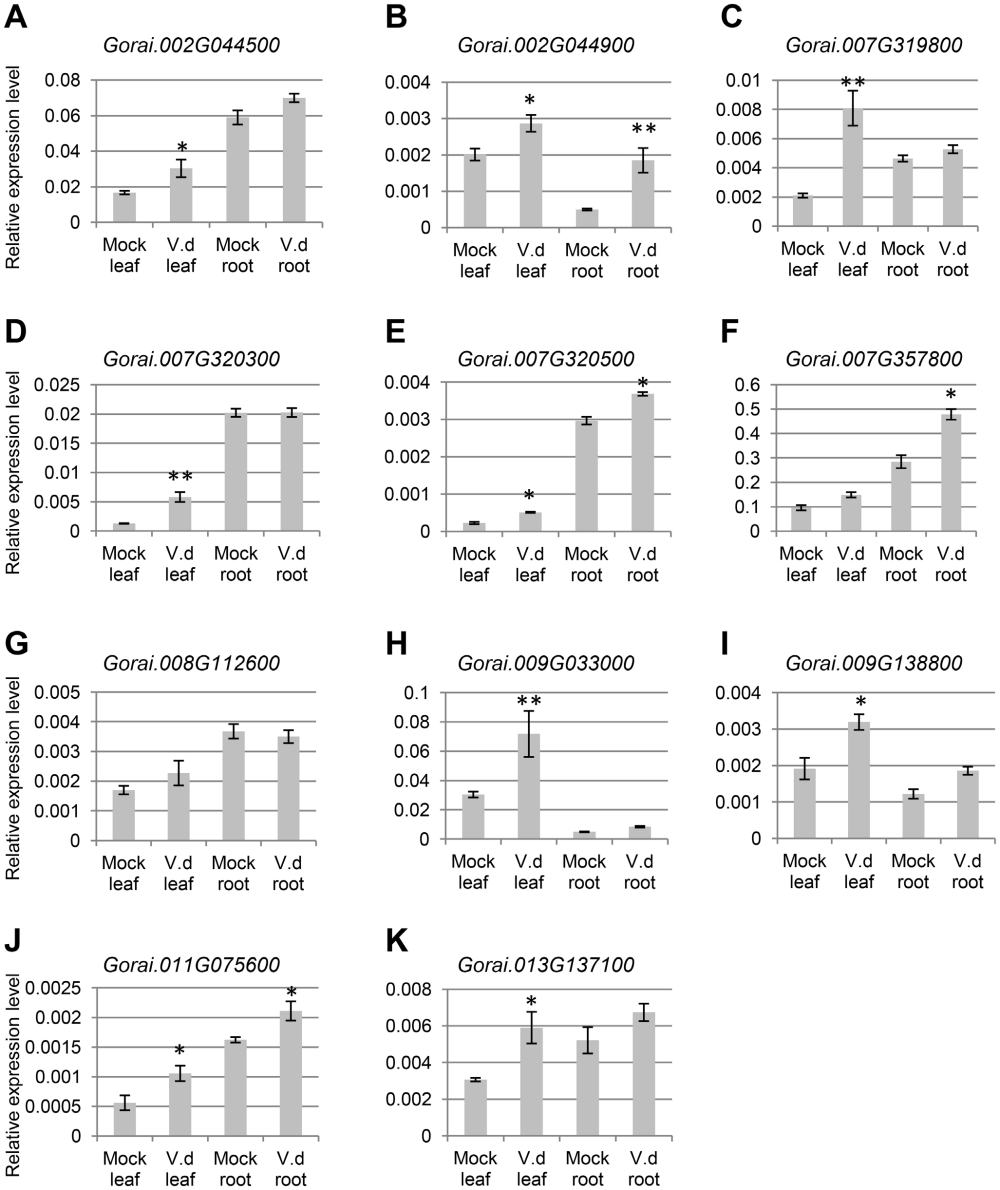
Expression analysis of *NBS-LRR* genes targeted by ghr-miR482/miR482.2. The same samples used in miRNA stem-loop qRT-PCR were used in this analysis. Error bars represent standard deviation of the expression ratio. * and ** denote significant relative to the corresponding mock-infected control at *p*<0.05 and *p*<0.01, respectively.

To know whether, like in tomato plants infected with viruses or bacteria, the expression levels of miR482 were down-regulated in cotton plants infected with fungal pathogen *V. dahliae* by root dipping (Figure S6), we first performed small RNA northern blots using RNA isolated from the susceptible Sicot71 cultivar (*G. hirsutum*). Expression of ghr-miR482a/miR482e.2 was detected in leaves collected at 1 dpi from plants infected with *V. dahliae* by root dipping, but no *V. dahliae-*induced down-regulation of ghr-miR482a/miR482e.2 was observed (Figure 6A). We were unable to detect expression of these two miRNAs in roots. We then performed the more sensitive miRNA stem-loop qRT-PCR to analyze the expression levels and changes of individual members of the ghr-miR482 family in response to *V. dahliae* infection. In leaves and roots, ghr-miR482b was the most abundantly expressed while ghr-miR482a was the least expressed under mock infection (Figures 6B–6H). Upon *V. dahliae* infection, a decrease in expression was observed for ghr-miR482b/miR482b.2 and ghr-miR482c in both leaves and roots, as well as for ghr-miR482d.2 in roots (Figures 6C, 6D, 6F, 6G), whereas the expression levels of ghr-miR482a, ghr-miR482d and ghr-miR482e.2 were unchanged (Figures 6B, 6E, 6H). To determine whether this down-regulation of ghr-miR482/miR482.2 caused up-regulation of their possible *NBS-LRR* targets, expression changes of 11 *NBS-LRR* genes that were predicted targets of ghr-miR482/miR482.2 were analysed. Ten of these 11 *NBS-LRR* genes were found to be induced upon *V. dahliae* infection in either leaves or roots, or both (Figure 7). Significant induction in both leaves and roots was observed for *Gorai.002G044900*, *Gorai.007G320500* and *Gorai.011G075600* (Figures 7B, 7E, 7J), and significant induction in leaves or roots was observed in seven genes (Figures 7A, 7C–D, 7F, 7H–I, 7K). These results suggest that the *NBS-LRR* target genes annotated in *G. raimondii* are conserved in *G. hirsutum*, and that an induced expression of *NBS-LRR* target genes in *V. dahlia*-infected *G. hirsutum* was a result of repression of ghr-miR482/miR482.2 biogenesis. *Gorai.008G112600*, a predicted target of ghr-miR482a-c (Table S1), remained unchanged (Figure 7G). One possibility is that the miR482-target sites of *Gorai.008G112600* and its *G. hirsutum* homolog are different and the latter is no longer a target of ghr-miR482a-c in *G. hirsutum*. Another possibility is that *Gorai.008G112600* is a target of ghr-miR482a, which was lowly expressed and not *V. dahlia*-infection responsive, rather than ghr-miR482b/c because it is a better target of ghr-miR482a based on prediction (Table S1). In addition, the possibility of translational repression also could not be ruled out.

## Discussion

miR482 is a diversified miRNA family whose abundance varies significantly among different plant species. It is highly expressed in *Solanaceae* species, but rarely detected in monocotyledonous species [12]. *Arabidopsis* has no miR482 annotated, but its miR472 is closely related to miR482 found in other species. Interestingly, some *MIR482* loci are able to produce a miR482.2 variant, whose 1^st^–20^th^ nucleotides are overlapping with the 3^rd^–22^nd^ nucleotides of miR482. This variant was designated miR2118 in some plant species [10]. In rice, miR2118 is highly expressed in developing inflorescence and triggers production of phased siRNAs [31], [32] but no miR482 has been reported. According to the published cotton small RNA data [10], [20], [21], [22], [26] we analysed, the miR482 family of *G. hirsutum* has at least four members, and two members (ghr-miR482b and ghr-miR482d) have a corresponding ghr-miR482.2. In addition, ghr-miR482e.2 but not its corresponding ghr-miR482e is found in the *G. hirsutum* small RNA datasets we analysed. By blasting the *G. raimondii* genome sequence, we found that ghr-miR482a has no homolog and thus could be a newly evolved ghr-miR482 member in *G. hirsutum*. Although ghr-miR482b and ghr-miR482c each have a single match in the *G. raimondii* genome, ghr-miR482d has five matches (gra-miR482d, f-i), suggesting that ghr-miR482d could be generated from multiple loci in the D_t_ genome of *G. hirsutum*. All seven members of the ghr-miR482 family (Figure 1) are found in *G. arboreum* [10], suggesting that they may also be expressed from the A_t_ genome of *G. hirsutum*. In addition, we identified at least five precursors of new isoforms of ghr-miR482d in the *G. raimondii* genome, but these isoforms were not found in the *G. hirsutum* small RNA datasets we analysed. It could be that these ghr-miR482d isoforms are expressed at very low levels or in tissues that have not been used in generating the small RNA data. Another possibility is that they have been lost in *G. hirsutum* after polyploidization.

miRNAs have been shown to play an important role in response to biotic and abiotic stresses in plants [3], [5], [6]. Several miRNAs have been demonstrated to be regulators of the pathways related to plant immunity. For example, miR393 was induced by flg22 to restrict *Pseudomonas syringae* growth by repressing auxin signalling [9]. A *Brassica*-specific miRNA, bra-miR1885, was specifically induced by infection with TuMV (turnip mosaic virus), but not with CMV (cucumber mosaic virus), TMV (tobacco mosaic virus) or *Sclerotinia sclerotinorium*, a necrotrophic fungal pathogen. Bra-miR1885 has been shown to cleave a TIR-NBS-LRR (TNL) type disease resistance gene [33], although the function for the pathogen induced expression of bra-miR1885 and cleavage of disease resistance gene is yet to be investigated.

Expression of miR482 was found to be suppressed in tomato plants infected with viruses or bacteria, and consequently some of its disease resistant *NBS-LRR* target genes were induced two to three-folds [12]. Based on this observation and on the experiment, in which *N. benthamiana* mRNAs encoding NBS-LRR proteins were found to be silenced by tomato miR482, the authors have proposed that plants are able to exploit virus- and bacterium-derived suppressors of RNA silencing to induce expression of defense-related genes to achieve non-race-specific resistance against viral and bacterial pathogens [12]. Disease resistance proteins may have a cost to plants [34] because if unregulated they can trigger autoimmunity in the absence of pathogen infection and inhibit plant growth [11]. Plants have thus evolved the miR482-*NBS-LRR* regulatory loop as a counter mechanism to minimize the cost of over-expression of *NBS-LRR* genes in the absence of a pathogen, and to ensure rapid induction of disease resistance proteins upon pathogen attack.

In this study, we asked the question whether the mechanism uncovered in tomato for viral and bacterial pathogens is conserved for fungal pathogen in cotton. We confirmed some of the predicted targets were cleaved by ghr-miR482/miR482.2 (Figure 3) and found that miR482-mediated cleavage of *NBS-LRR* genes triggers production of phased siRNAs (Figures 5, S5). Furthermore we found that *V. dahliae* infection resulted in down-regulation of some members of the ghr-miR482 family and up-regulation of their *NBS-LRR* targets (Figures 6–7). Individual members of the miR482 family behaved differently in response to *V. dahliae* infection. Three members remained unchanged and the highest expressed and significantly down-regulated member, ghr-miR482b, seems to have no contribution towards the induction of the *NBS-LRR* genes examined. The exact reason for this still needs further investigation, but ghr-miR482b has the least number of predicted *NBS-LRR* targets and has no unique target. Of the 36 predicted *NBS-LRR* targets, only two are targets of ghr-miR482b and both are better targeted by other members of the ghr-miR482 family (Table S1).

The miR482-mediated regulation of *NBS-LRR* genes seems to be finely modulated in a few different ways. The target site (P-loop) of miR482 is one of the conserved motifs in the NBS-LRR proteins. The miR482 family is thus expected to regulate the expression level of a number of *NBS-LRR* genes. We found that ∼12% of *NBS-LRR* genes in the *G. raimondii* genome are potential targets of the miR482 family, and that 10 of the 11 analysed could be induced upon *V. dahliae* infection. On the other hand, the number of *NBS-LRR* genes regulated by miR482 in each species could be evolutionally determined by the balance between minimizing the disadvantageous effect of over-expression of *NBS-LRR* genes in the absence of a pathogen and maximizing the induction of *NBS-LRR* genes in the presence of a pathogen. This is supported by the observation that both the miR482 sequences and the predominant amino acid sequences of the P-loops of NBS-LRR proteins in cotton are different from those in tomato, soybean and *M. truncatula* [10], [12], a result of co-evolution of miR482 and their potential *NBS-LRR* targets in each species. Gain and loss of regulation of *NBS-LRR* genes by miR482 could be an on-going evolutionary event because for each type of P-loop the nucleotide variation was always found at the synonymous 3^rd^ position of a codon (Figure 4). Furthermore, diverse and finely controlled biogenesis of miR482/miR482.2 seems to play a role in the modulation of miR482-mediated regulation of *NBS-LRR* genes. Some *MIR482* loci generate both miR482 and its variant miR482.2, and some generate only either miR482 or miR482.2. In addition, gra-miR482h/miR482h.2 and gra-miR482e.2 on scaffold 9 are contained in a single transcript (Figure 2A).

When we were performing northern blot analysis, we assumed that the overall expression level of all members of the ghr-miR482 family could be detected by using one representative member of ghr-miR482 and ghr-miR482.2 (ghr-miR482a and ghr-miR482e.2 used), respectively, as probe. In view of the expression level of ghr-miR482b and its significant reduction in the *V. dahliae-*infected leaves (Figure 6C), this assumption seems to be incorrect. There are three mismatches between ghr-miR482a and ghr-miR482b/miR482d, although only one mismatch between ghr-miR482a and ghr-miR482c. Four mismatches are present between ghr-miR482e.2 and ghr-miR482b.2, and six between ghr-miR482e.2 and ghr-miR482d.2 (Figure 1). Therefore, the signal detected by northern blot might be only for ghr-miR482a and ghr-miR482e.2, a result consistent with that of miRNA stem-loop qRT-PCR (Figures 6B, 6H).

In conclusion, on the basis of characterization of the miR482 family and identification of *NBS-LRR* genes targeted by ghr-miR482/miR482.2, we demonstrated that *V. dahliae* infection represses certain members of the miR482 family and induces expression of specific disease resistance *NBS-LRR* genes in cotton. This suggests that the miR482-*NBS-LRR* regulatory loop is part of the immune responses induced not only by viral and bacterial pathogens but also by fungal pathogens.

## Materials and Methods

### Plant Materials and *Verticillium Dahliae* Inoculation

*G. hirsutum* (varieties Sicot71 and Texas Marker-1, or TM-1) and *G. arboreum* (variety Yunnanzhongmian, or YZ) plants were raised from seeds and grown in glasshouse at 28±2°C with 16 hrs day and 8 hrs night regime. One-true-leaf whole seedlings (including roots) of TM-1 and YZ were used in DNA isolation. The same stage seedlings of Sicot71 were used in *V. dahliae* inoculation by root dipping. This was done by submerging the roots of cotton plants into a suspension of *V. dahliae* conidia for 5 minutes. The inoculated plants were then planted in soil in 8-cm pots. The *V. dahliae* inoculum was prepared by growing *V. dahliae* in potato dextrose broth (PDB, 1/2 strength) on a shaker (25°C) for 9 days. The conidial suspension was diluted to ∼10^8^ spores/ml with full strength PDB before inoculation. Leaf and root samples were separately collected from *V. dahliae* infected and mock-treated (dipping in water) plants at one day-post-inoculation (dpi).

### Small RNA Datasets and Phased Small RNA Analysis

Previously published small RNA datasets, including GSM699074–699077 [26], GSM717570–717572 [10], GSM634227–634228, GSM686014–686015 and GSE16332 [21], generated using various cotton tissues from different cotton species were downloaded from http://www.ncbi.nlm.nih.gov/geo/and used in identification of miR482 and miR482.2. Of these datasets, GSM699074–699077 were aligned to the *G. raimondii* genome sequence [24] for identification of *NBS-LRR* genes generating phased secondary small RNAs, which was performed according to the approach described previously with a phase score >2 [17], [30].

### Identification of *MIR482* in the *G. raimondii* Genome

*G. raimondii* short sequences with 0–1 mismatch in comparison to their closest member of the miR482 family of *G. hirsutum* were first identified using each ghr-miR482 or ghr-miR482.2 as a query. Two-hundred base pairs of *G. raimondii* sequence flanking each identified short sequence were then retrieved and subjected to hairpin structure prediction using RNA-fold. A *MIRNA-like* hairpin contains a ghr-miR482, ghr-miR482.2 or their isoform was considered as *MIR482* or *MIR482.2* of *G. raimondii*.

### Identification of Gra-miR482 Targets in the *G. raimondii* Genome and Confirmation of ghr-miR482/miR482.2-mediated Cleavage of *NBS-LRR* Genes

Putative targets of gra-miR482/miR482.2 were first predicted based on the annotated *G. raimondii* transcripts [24] using psRNATarget (http://plantgrn.noble.org/psRNATarget/) [35], and were then manually checked and selected based on the following criteria: no mismatch (except G::U pair) at positions 10 and 11 (relative to the 5' end of miRNA); no two consecutive mismatches but one mismatch flanked by a G::U pair allowed; with a score ≤3.5 (based on 1 for mismatch and G::U pair at position 10 or 11; 0.5 for G::U pair at positions other than positions 10 and 11).

Confirmation of ghr-miR482/miR482.2-mediated cleavage of *NBS-LRR* genes was carried out using total RNA by 5′ RACE (rapid amplification of cDNA ends) as previously described [36]. Briefly, ∼2 µg of RQ1 DNase (Promega) treated total RNA isolated from Sicot71 was ligated with 100 pmol of the 5′ adaptor (5' AACAGACGCGUGGUUACAGUCUUG 3') using T4 RNA ligase (NEB) in the presence of 1 mM of ATP and 1 µl of RNaseOUT (Invitrogen). Two rounds (primary and nested) of PCR were performed using gene specific reverse primers and a universal forward primer (GUS_RACEf; Table S2) that annealing to the 5′ adaptor. Bands with an expected size were gel purified using the MinElute® Gel Extraction Kit (Qiagen) and inserted into pCR®4-TOPO® vector (Invitrogen). At least four clones were sequenced for each ligation using the T7 primer.

### Northern Blot and qRT-PCR

Approximately 25 µg of total RNA isolated using the hot borate approach [37] was used in small RNA northern blot analysis as previously described [36]. The antisense sequences of ghr-miR482a and ghr-miR482e.2 were used as probes. Because of similar sequences between miR482 and miR482.2, we reasoned that the expression of miR482 and miR482.2 could not be unambiguously detected by the miR482 and the miR482.2 antisense probe, respectively; therefore, both probes were used together. miR482 stem-loop qRT-PCR was performed according to the approach reported previously [38]. The expression levels of *NBS-LRR* genes in *V. dahliae* infected and mock-treated cotton leaves and roots were analysed as previously described [39]. Briefly, reverse transcription was performed using 1 µg of RQ1 DNase (Promega) treated total RNA, random primer or stem-loop RT primer, and SuperScript III reverse transcriptase (Invitrogen). The first-strand cDNA reaction was diluted 10 folds prior to qPCR and 4.6 µl of the diluted cDNA was then used as the PCR template. Reverse transcriptase negative controls were performed for each reverse transcription (RT) reaction to make sure there is no genomic DNA contamination. Each miRNA or gene was analysed using three biological replicates each with three technical replicates. Relative expression levels of the target genes were calculated using 2^−ΔCt^. Significance was analysed by *t*-test. Cotton Histone 3 (Accession no. AF024716) was used as the reference for normalization [21]. All qRT-PCR reactions were run on the ABI PRISM™ 7900HT Fast Real-Time PCR System (ABI) using SYRB® GreenER™ qPCR SuperMix (Invitrogen). Oligos used in northern blot and qRT-PCR analyses are shown in Table S2.

## Supporting Information

Figure S1 **Precursors of the members of the gra-miR482 family.**
(TIF)

Figure S2 **Precursors of the isoforms of gra-miR482d and gra-miR482f-i (one mismatch with gra-miR482d, f-i).**
(TIF)

Figure S3 **Alignment of the precursor sequence of gra-miR482h/miR482h.2 and gra-miR482e.2 with the tentative consensus sequence TC257237 (1446 bp).**
(TIF)

Figure S4 **Hairpin structure of the region containing miRCan1 and miR482e.2 in different genomes.**
(TIF)

Figure S5 ***NBS-LRR*** **genes generating phased secondary small RNAs.**
(TIF)

Figure S6 **Comparison of cotton seedlings (** ***G. hirsutum*** **, cv Sicot71) inoculated with** ***Verticillium dahliae*** **(right pot) and mock treated (left pot).**
(TIF)

Table S1 **Predicted targets of miR482/miR482.2 and miRCan1 in** ***G. raimondii*** **.**
(XLSX)

Table S2 **Oligos used in this study.**
(DOC)
