# Supplementary figures and images for: miR482 Regulation of NBS-LRR Defense Genes during Fungal Pathogen Infection in Cotton
Source: PLoS One. 2013 Dec 31;8(12):e84390. doi: 10.1371/journal.pone.0084390 (PMC3877274; doi:10.1371/journal.pone.0084390)

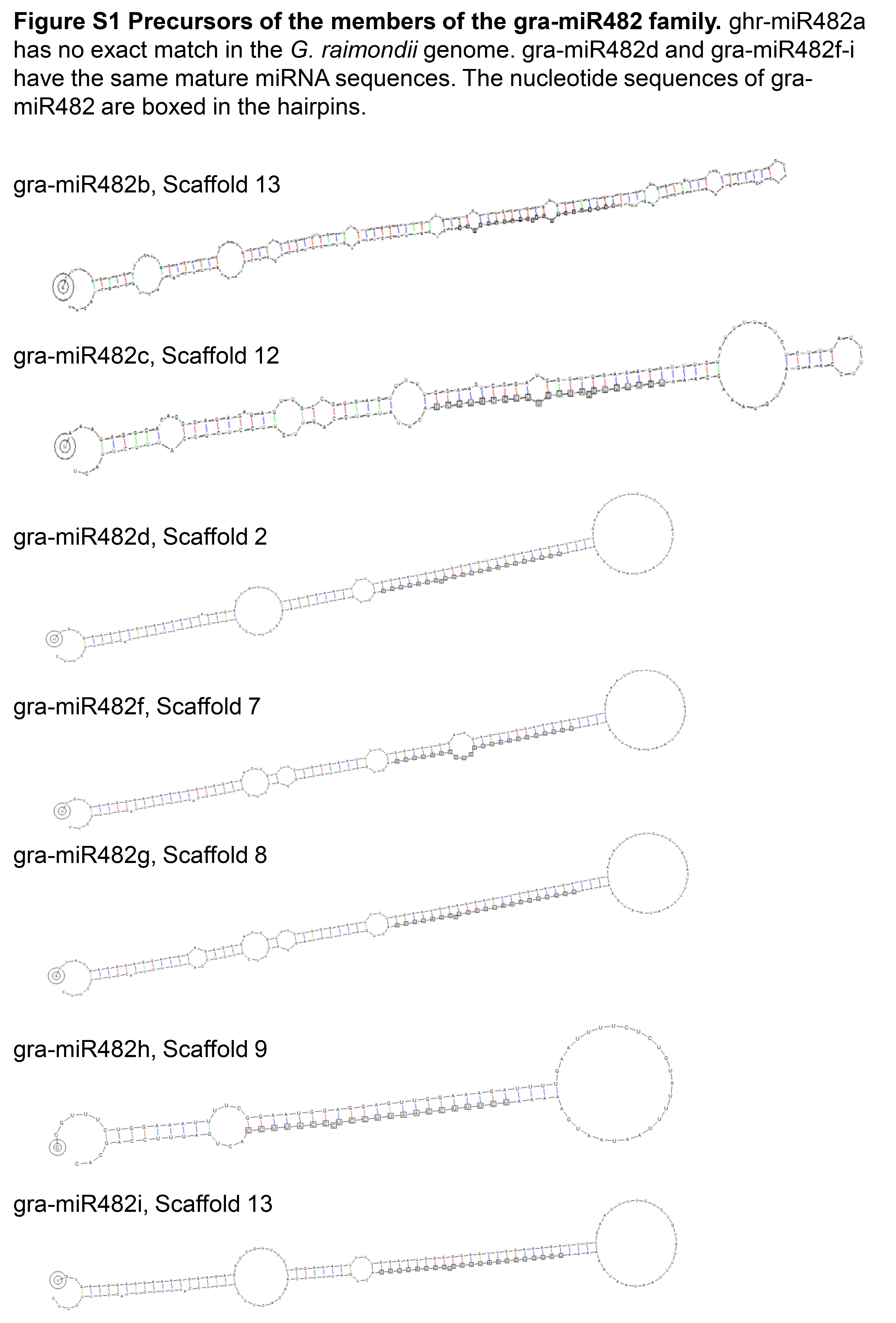

Supplement: Figure S1 — Precursors of the members of the gra-miR482 family. (TIF) [file pone.0084390.s001.tif]

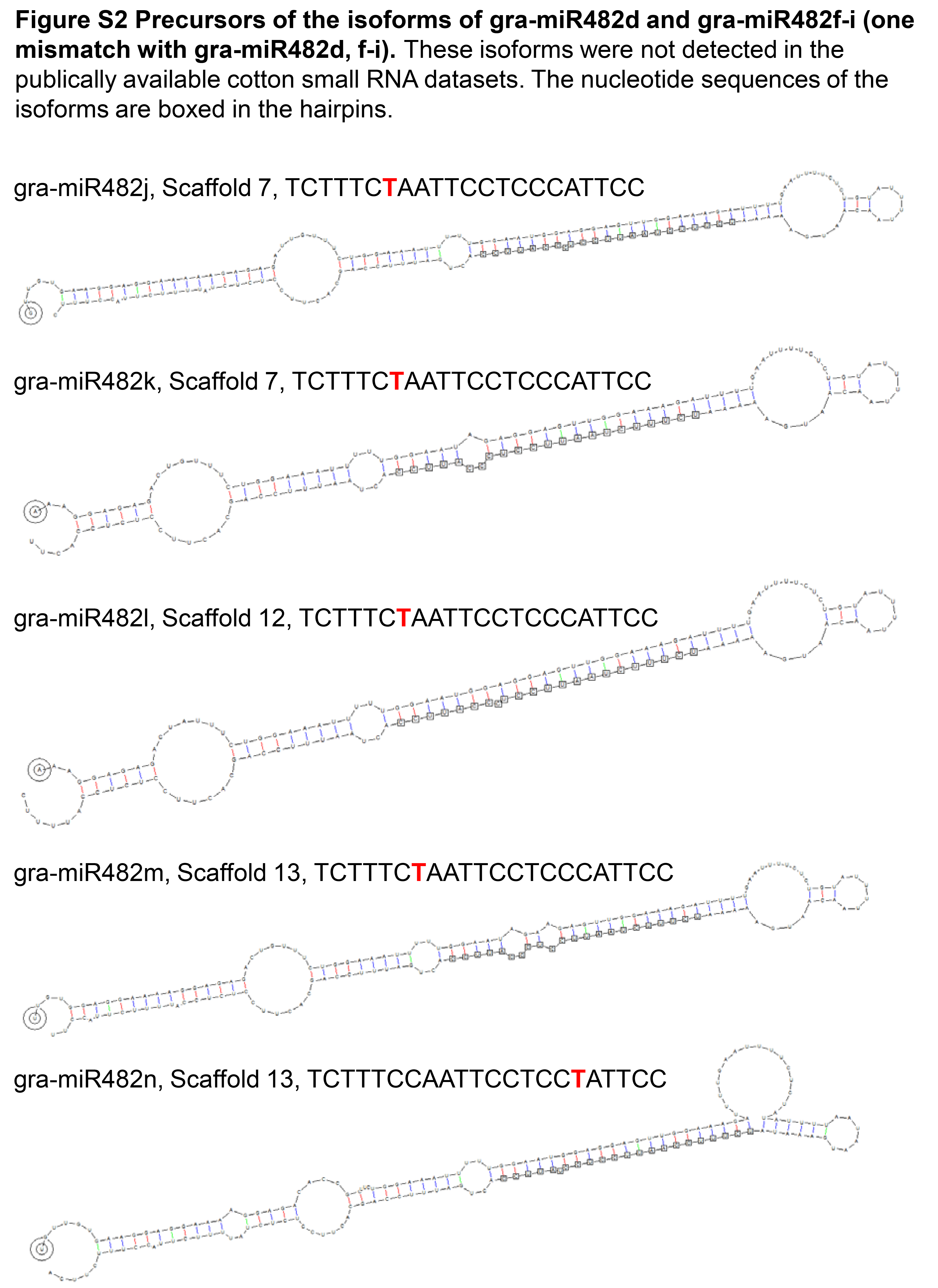

Supplement: Figure S2 — Precursors of the isoforms of gra-miR482d and gra-miR482f-i (one mismatch with gra-miR482d, f-i). (TIF) [file pone.0084390.s002.tif]

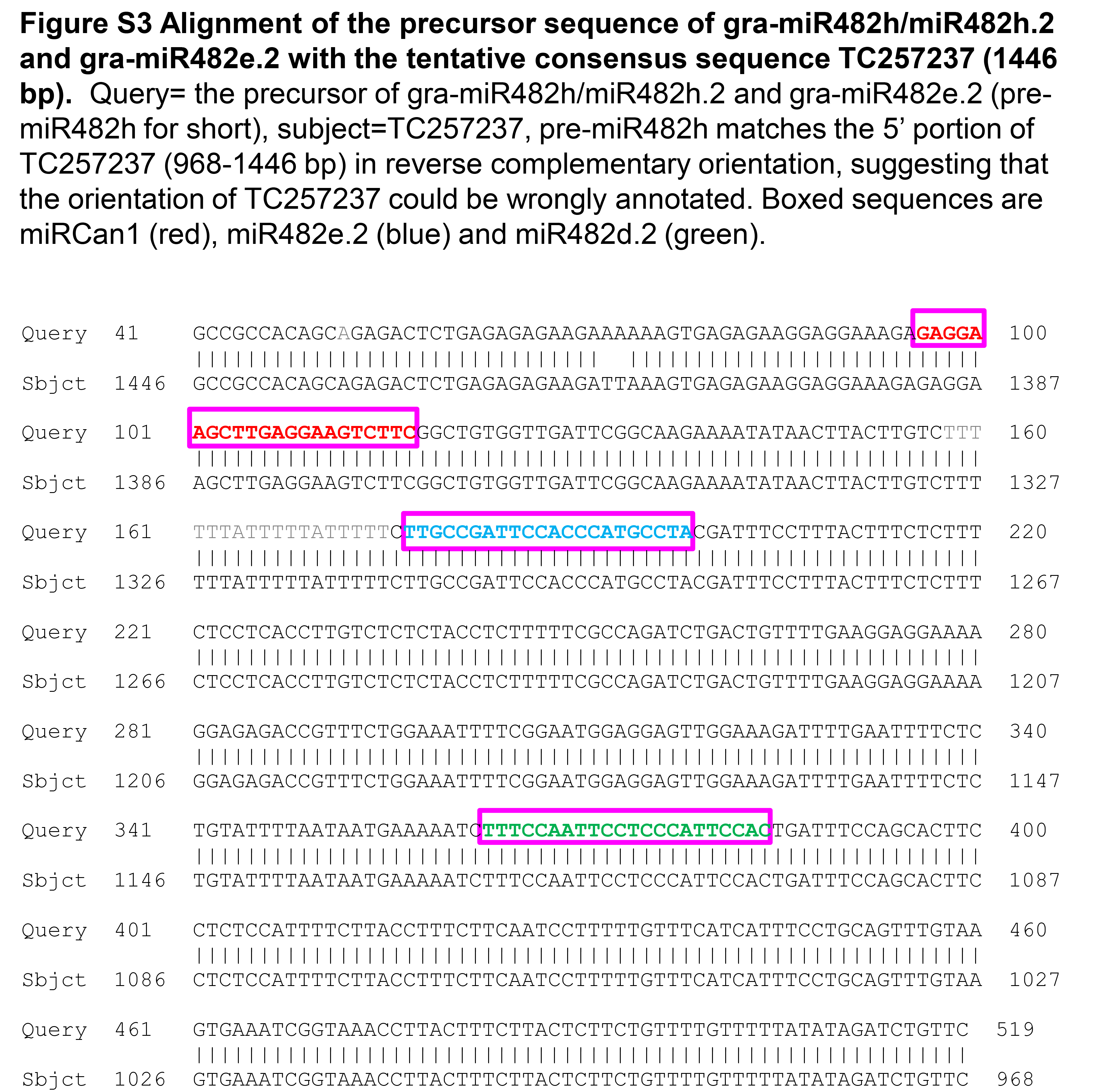

Supplement: Figure S3 — Alignment of the precursor sequence of gra-miR482h/miR482h.2 and gra-miR482e.2 with the tentative consensus sequence TC257237 (1446 bp). (TIF) [file pone.0084390.s003.tif]

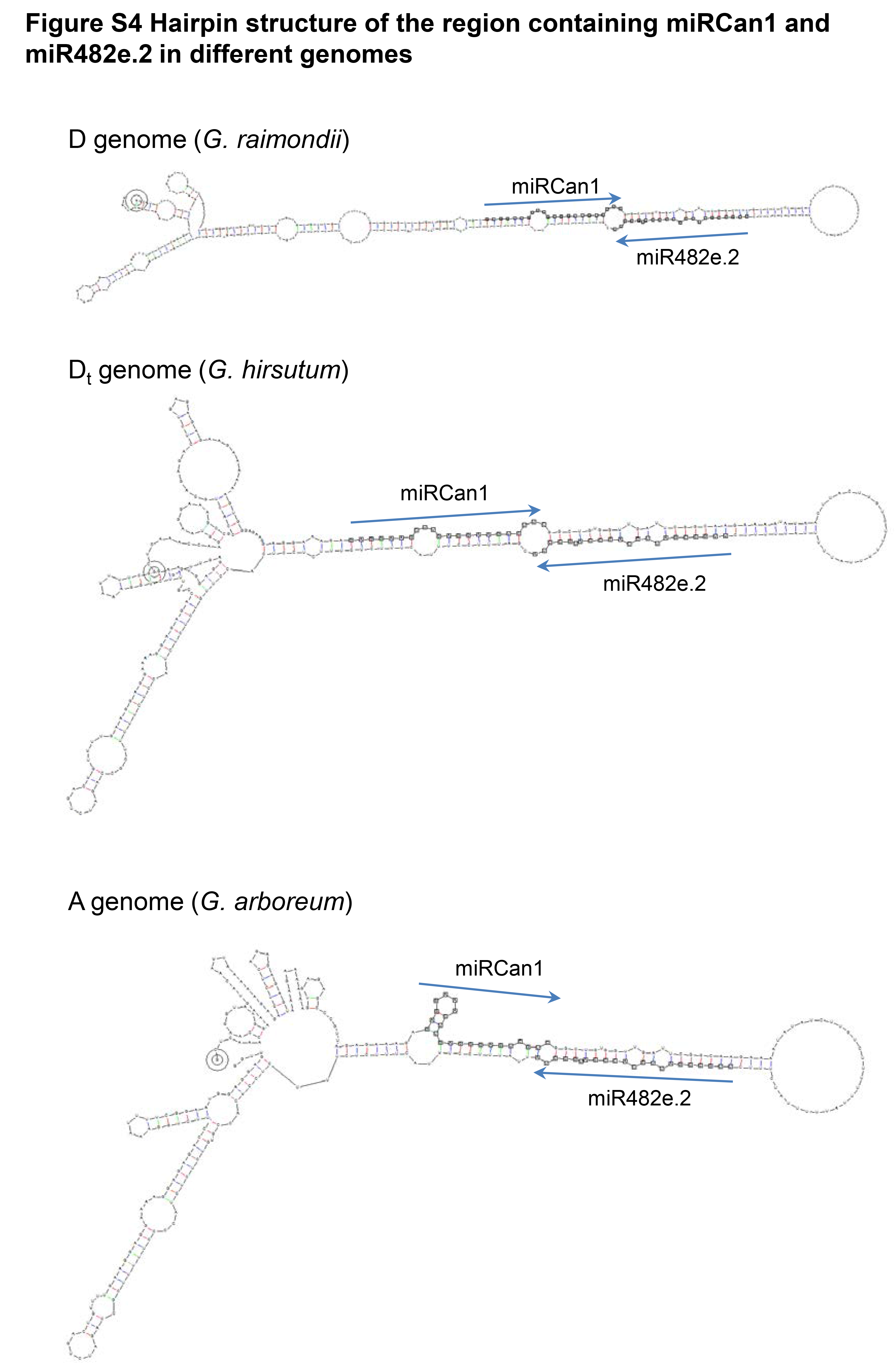

Supplement: Figure S4 — Hairpin structure of the region containing miRCan1 and miR482e.2 in different genomes. (TIF) [file pone.0084390.s004.tif]

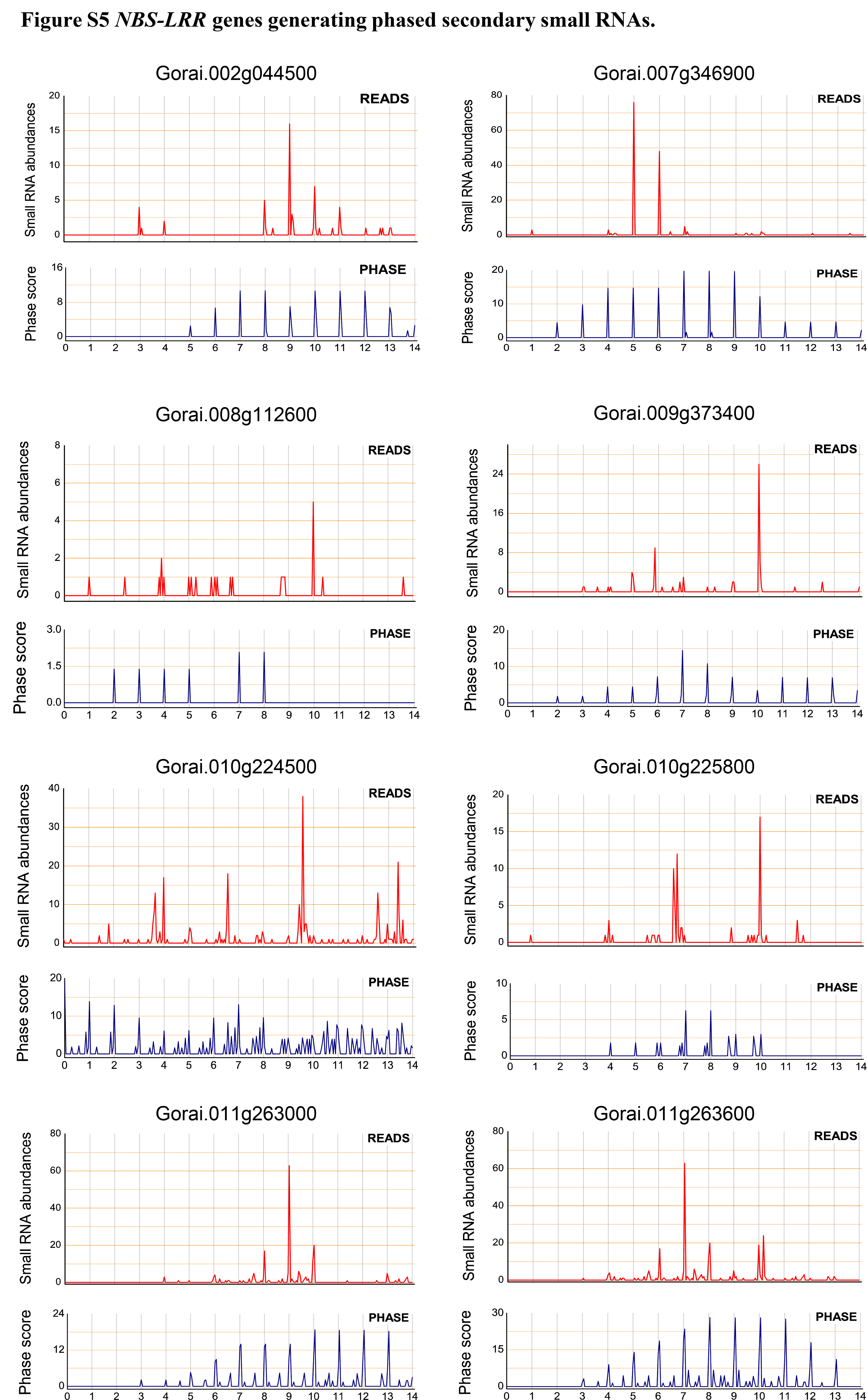

Supplement: Figure S5 — NBS-LRR genes generating phased secondary small RNAs. (TIF) [file pone.0084390.s005.tif]

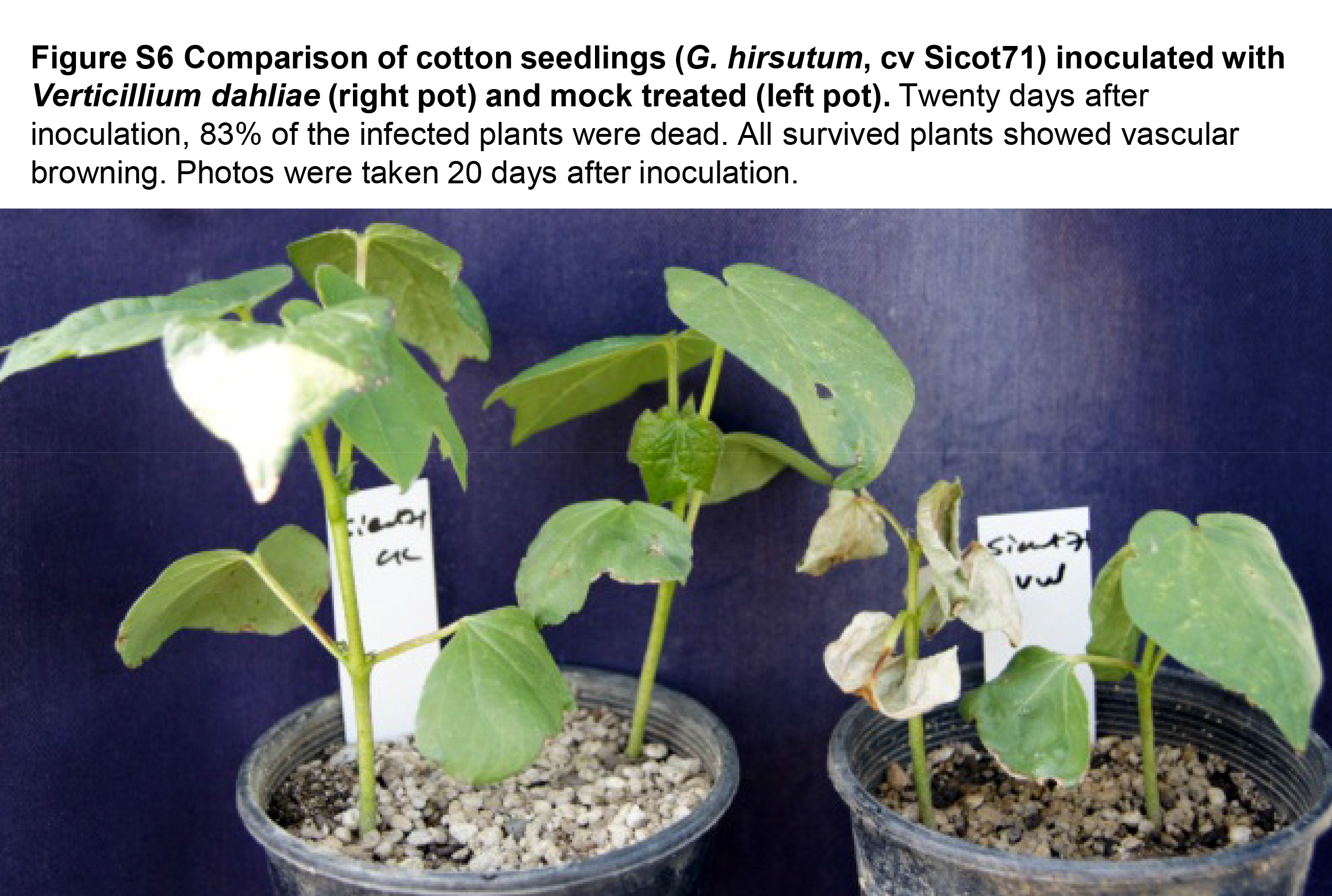

Supplement: Figure S6 — Comparison of cotton seedlings ( G. hirsutum , cv Sicot71) inoculated with Verticillium dahliae (right pot) and mock treated (left pot). (TIF) [file pone.0084390.s006.tif]
