# Supplementary material for: miR482 Regulation of NBS-LRR Defense Genes during Fungal Pathogen Infection in Cotton
Source: PLoS One. 2013 Dec 31;8(12):e84390. doi: 10.1371/journal.pone.0084390 (PMC3877274; doi:10.1371/journal.pone.0084390)
Supplement: Table S2 — Oligos used in this study. (DOC) [file pone.0084390.s008.doc]

**Table S2** Oligos used in this study

| Oligos | Sequence (5' - 3') | Usage |
| --- | --- | --- |
| Ghr-miR482a_as | GGTATGGGAGGAGTAGGAAAGA | Northern blot |
| Ghr-miR2118e_as | TAGGCATGGGTGGAATCGGCAA | Northern blot |
| Gorai.002G044500_F | GGAGGAGACTAAATCTAAACC | qRT-PCR |
| Gorai.002G044500_R | CCTGGTGATGTTGAAGTCCTG | qRT-PCR |
| Gorai.002G044900_F | GCACATACACGATCAACTATT | qRT-PCR |
| Gorai.002G044900_R | CAGTGACCCAAATGATTTTCT | qRT-PCR |
| Gorai.007G319800_F | GGGTCACTGTATCGAGGGAAT | qRT-PCR |
| Gorai.007G319800_R | GCCTCTGCAATCTTCTTCTG | qRT-PCR |
| Gorai.007G320300_F | GAGACCCACAAGTTCGATGTT | qRT-PCR |
| Gorai.007G320300_R | AGCAAGACTCATCTCCTTTG | qRT-PCR |
| Gorai.007G320500_F | GGGCAGGGATGTTGTTTGAA | qRT-PCR |
| Gorai.007G320500_R1 | CCTTCTCCCATACATCGTCCAAG | qRT-PCR |
| Gorai.007G357800_F | CTCTTGCTCAGCTTGTTTAC | qRT-PCR |
| Gorai.007G357800_R | CACATACCCAAGCCTTGAGAT | qRT-PCR |
| Gorai.008G112600_F | GAGAGCTTGGGTTTGTGTCT | qRT-PCR |
| Gorai.008G112600_R2 | AGGTCTTTGTCACCCTCAGCA | qRT-PCR |
| Gorai.009G033000_F | GATGTGGTAATATGGGTCTTG | qRT-PCR |
| Gorai.009G033000_R | TGGACTGTTGGATCTTACTAA | qRT-PCR |
| Gorai.009G138800_F | GCCCAATTAGCCTACAATGAC | qRT-PCR |
| Gorai.009G138800_R | CCGAGACGCACACCCATATTC | qRT-PCR |
| Gorai.011G075600_F | CGACACTTGCTCAACTGGTTTAC | qRT-PCR |
| Gorai.011G075600_R | GGGTCTGAGACACATATCCAT | qRT-PCR |
| Gorai.013G137100_F | CAAGACACTTCGACTGCCAT | qRT-PCR |
| Gorai.013G137100_R | GGTCCTTAACAACACCTCCAC | qRT-PCR |
| Histone 3_F | TCAAGACTGATTTGCGTTTCCA | qRT-PCR |
| Histone 3_R | GCGCAAAGGTTGGTGTCTTC | qRT-PCR |
| miR482a_SLR | GTCGTATCCAGTGCAGGGTCCGAGGTATTCGCACTGGATACGACGGTATG | Stem-loop RT |
| miR482b_SLR | GTCGTATCCAGTGCAGGGTCCGAGGTATTCGCACTGGATACGACGGCATG | Stem-loop RT |
| miR482cd_SLR | GTCGTATCCAGTGCAGGGTCCGAGGTATTCGCACTGGATACGACGGAATG | Stem-loop RT |
| miR2118b_SLR | GTCGTATCCAGTGCAGGGTCCGAGGTATTCGCACTGGATACGACGTGGCA | Stem-loop RT |
| miR2118d_SLR | GTCGTATCCAGTGCAGGGTCCGAGGTATTCGCACTGGATACGACGTGGAA | Stem-loop RT |
| miR2118e_SLR | GTCGTATCCAGTGCAGGGTCCGAGGTATTCGCACTGGATACGACTAGGCA | Stem-loop RT |
| miR482ac_SLR_F | GCGGCGTCTTTCCTACTCCTCC | Stem-loop qPCR |
| miR482b_SLR_F | GCGTCGTCTTGCCTACTCCACC | Stem-loop qPCR |
| miR482d_SLR_F | GCGGCGTCTTTCCAATTCCTCC | Stem-loop qPCR |
| miR2118b_SLR_F | GCTGCGTTGCCTACTCCACCCA | Stem-loop qPCR |
| miR2118d_SLR_F | GCGGCTTTTCCAATTCCTCCCA | Stem-loop qPCR |
| miR2118e_SLR_F | GCTGAGTTGCCGATTCCACCCA | Stem-loop qPCR |
| SLR_R | GTGCAGGGTCCGAGGT | Stem-loop qPCR |
| GUS_RACEf | AACAGACGCGTGGTTACAGTC | 5’ RACE |
| Gorai.002G044500_R1 | CTGCAATGTCTTGTTGTAACCTGGTG | 5’ RACE |
| Gorai.002G044500_R2 | CCTGGTGATGTTGAAGTCCTGTGAAA | 5’ RACE |
| Gorai.007G319800_R1 | CCCTGCTCTTAATATTTCATCTTCATCTACGG | 5’ RACE |
| Gorai.007G319800_R2 | CCCTCGATACAGTGACCCATAC | 5’ RACE |
| Gorai.007G320500_R1 | CCTTCTCCCATACATCGTCCAAG | 5’ RACE |
| Gorai.007G320500_R2 | CCCTGCCCTTGTTGTTTCACATTC | 5’ RACE |
| Gorai.009G033000_R1 | CCCCCAGATCAATTCCCTCCCAA | 5’ RACE |
| Gorai.009G033000_R2 | GGAGTTCATGGGCTTCACTATCTGC | 5’ RACE |
